# Splitting Choice and Computational Complexity Analysis of Decision Trees

**DOI:** 10.3390/e23101241

**Published:** 2021-09-24

**Authors:** Xin Zhao, Xiaokai Nie

**Affiliations:** 1 School of Mathematics, Southeast University, Nanjing 211189, China; 2 School of Automation, Southeast University, Nanjing 210096, China; xnie@seu.edu.cn

**Keywords:** decision tree, splitting bias, splitting criteria, computational complexity, noise variable

## Abstract

Some theories are explored in this research about decision trees which give theoretical support to the applications based on decision trees. The first is that there are many splitting criteria to choose in the tree growing process. The splitting bias that influences the criterion chosen due to missing values and variables with many possible values has been studied. Results show that the Gini index is superior to entropy information as it has less bias regarding influences. The second is that noise variables with more missing values have a better chance to be chosen while informative variables do not. The third is that when there are many noise variables involved in the tree building process, it influences the corresponding computational complexity. Results show that the computational complexity increase is linear to the number of noise variables. So methods that decompose more information from the original data but increase the variable dimension can also be considered in real applications.

## 1. Introduction

Decision trees [1,2,3] are a decision support tool that use a tree-like graph or model of decisions either for classification or regression. Both classification trees and regression trees can be seen as supervised learning models, the former one maps the input space into predefined classes while the latter one maps the input space into a real-valued domain. As an important part of data mining, decision trees are a discovery and prediction-oriented supervised inductive learning method in which the trained model is assumed to be applicable to future, unseen, examples. The meaning of classification not only includes identifying which group a new observation belongs to, on the basis of training dataset, but also includes learning how this new observation is identified by detecting the variables’ difference between groups. In most cases, both identifying and learning are important, but sometimes, learning is more important when the class has already been provided. Similarly, for regression trees, the aim is to predict the new observation’s response variable value and understand how it is determined.

The method decision trees have many advantages that others do not have [4,5,6,7]. Decision trees can discover the hidden decision rules, which have quite high interpretability in explaining real applications. There are also many criteria to choose under different situations, thus leading to more possibility in modeling. For learning different variables’ behavior between different groups, many traditional methods test variables’ values to determine whether they differ significantly or not across different groups, typically using means and variances. Subtle trends, however, may not be detected. So more complex statistical models, like logistic regression [8,9], can be built to explore the information involved in the data, but usually require many assumptions to make parameter estimation possible. For example, logistic regression requires the observations to be independent of each other and for there to be little or no multicollinearity among the independent variables. If the assumptions are not valid, solutions obtained from these methods are not reliable. In practice, some variables are correlated with each other. These are typically against the assumptions required and will inevitably lead to unreliable results.

These advantages undoubtedly bring convenience to decision making in medicine [10,11], commerce [12,13], and elsewhere. Classification and regression trees (CART) proposed by Loh [14] are one type of decision tree. This model splits the original dataset recursively using the Gini index [15], twoing criteria [14] or ANOVA [16,17] to decide which variable is most important and continues growing the tree until some criteria are achieved. It can output a variable importance list and the corresponding accuracy. We have applied decision trees to classification and regression problems in Zhao et al. [18] and Zhao et al. [19] and find good behavior in these applications.

However, CART has some undesirable properties like tending to select variables that have many classes (values) or many missing values. Different variables have different properties. For example, some categorical variables only have two possible values while some have a lot. Variables with many missing values maybe collected under low collection frequency. Different properties of the variables may cause bias in the modeling process. CART may favor some kinds of variables. In this research, the influence of different property is explored. Specifically, the following areas are explored: the splitting bias due to missing values under two different conditions and due to more values or categories (Section 2), and the influence of noise variables on computational complexity (Section 3). Some conclusions and future research are shown in Section 4.

## 2. Splitting Bias

In this section, the properties of different splitting criteria (entropy, Gini, etc.) are explored under different conditions. The splitting bias is defined as the difference between the observed and the theoretical information gain. For classification trees, one of the most popular criteria is information gain, namely the Shannon entropy information gain from parent node to child nodes. However this criterion is liable to unfairly favor attributes with large numbers of values or categories compared to those with few. This will be proven later in this section. In this sense, noise variables with large numbers of values could be selected in preference to genuinely informative attributes with fewer values. In general, this would lead to poorer predictive performance from the resulting tree. The probability to choose predictor variables with more information decreases.

In addition, splitting rules favor those noisy predictor variables with more missing values since their sample size is smaller than others. In this case, as the sample size decreases, the probability for choosing noisy predictor variables with more information decreases.

The gain ratio calculated from information gain also suffers the same kind of problem. It is acknowledged that attributes with very low information values (low attribute information) appear to gain an unfair advantage [20].

Another splitting criterion is $\chi^{2}$. In fact, this criterion is not biased since for different degrees of freedom, $\chi^{2}$ follows different probability distribution functions. Using degrees of freedom, $\chi^{2}$ eliminates the problem of bias. Although there are splitting criteria like $\chi^{2}$ that have no bias, CHAID [21] in R, which uses $\chi^{2}$ as the splitting criterion, however requires dependent and explanatory variables both to be categorical variables, which is not suitable for the datasets. For regression problems, ctree [2,3] will be used, which is an unbiased method, having no splitting bias in these cases.

### 2.1. Bias Due to Missing Values

In this section, it will be shown that both Gini and entropy information have bias in favor of choosing variables with more missing values. So no matter which splitting criterion is chosen, we have to face the bias due to missing values. That is why pre-processing is applied to missing values in the real data application.

When information gain is calculated, there is a bias between the theoretical gain and observed gain values due to the difference between the sample and population distributions. This bias can be different when there are missing values. For missing values in independent variables, most procedures deal with them by leaving out incomplete observations. The models in this research actually are more ambitious. Any observation with values for the dependent variable and at least one independent variable will participate in the modelling process [22]. For the Gini index, how bias is influenced by missing values has been investigated by Strobl et al. [20]. So an equivalent analysis for entropy is conducted as the following.

Assume there are an independent variable *X* and a dependent variable *Y* with two categories. The number of observations in the first category for *Y* is $N_{1}$, and that in the second category is $N_{2}$, with a summation as *N*. Then the entropy information for the root node is

$$ent_{N}=-\frac{N_{1}}{N}\log_{2}\left(\frac{N_{1}}{N}\right)-\frac{N_{2}}{N}\log_{2}\left(\frac{N_{2}}{N}\right).$$

In order to calculate the expectation of $ent_{N}$, for simplicity, we first calculate the bias for $E\left(\frac{N_{2}}{N}\log_{2}\left(\frac{N_{2}}{N}\right)\right)$, where $N_{2}∼B\left(N,p\right)$ and *N* is fixed. Specifically, *B* denotes binomial distribution, N denotes the total number of observations, p denotes the probability p=P(Y=secondcategory). The result is

$$\begin{array}{cc} E\left(-\frac{N_{2}}{N}\log_{2}\left(\frac{N_{2}}{N}\right)\right)= & E\left(-\frac{N_{2}}{N}\left(\log_{2}\left(N_{2}\right)-\log_{2}\left(N\right)\right)\right)\\ = & E\left(-\frac{N_{2}}{N}\left(\log_{2}\left(N_{2}\right)\right)\right)+p\log_{2}\left(N\right). \end{array}$$

If bias has value 0, that is the observed information gain is equal to the theoretical information gain, then

$$E\left(-\frac{N_{2}}{N}\log_{2}\left(\frac{N_{2}}{N}\right)\right)=-p\log_{2}\left(p\right),$$

so that

$$E\left(-\frac{N_{2}}{N}\left(\log_{2}\left(N_{2}\right)\right)\right)=-p\log_{2}\left(Np\right).$$

Then bias is given by $E\left(-\frac{N_{2}}{N}\left(\log_{2}\left(N_{2}\right)\right)\right)-\left(-p\log_{2}\left(Np\right)\right)$. Similarly, we can get the bias for $N_{1}$, which follows $B\left(N,1-p\right)$. Then the total bias for the root node is

$$\begin{array}{cc} E\left(bias_{N}\right)= & E\left(-\frac{N_{1}}{N}\left(\log_{2}\left(N_{1}\right)\right)\right)-\left(-\left(1-p\right)\log_{2}\left(N\left(1-p\right)\right)\right)\\ \; & +E\left(-\frac{N_{2}}{N}\left(\log_{2}\left(N_{2}\right)\right)\right)-\left(-p\log_{2}\left(Np\right)\right). \end{array}$$

It is not easy to get $E\left(-\frac{N_{1}}{N}\left(\log_{2}\left(N_{1}\right)\right)\right)$ and $E\left(-\frac{N_{2}}{N}\left(\log_{2}\left(N_{2}\right)\right)\right)-\left(-p\log_{2}\left(Np\right)\right)$ analytically as they contain the terms of the form $E\left(N_{1}\log_{2}\left(N_{1}\right)\right)$, so a polynomial expression is used to approximate the log function. Given that

(1) $$log_{2}\left(1+a\right)=a-\frac{a^{2}}{2}+\frac{a^{3}}{3}\cdots \;\;,$$

for |a|<1, we substitute a=p−1 in Equation (1), and require that *p* is not small. If $X∼B\left(n,p\right)$, then its moments are given by

$$\begin{array}{cc} E\left(X\right) & =np,\\ E\left(X^{2}\right) & =np+n\left(n-1\right)p^{2},\\ E\left(X^{3}\right) & =np+p^{2}\left(3n^{2}-3n\right)+p^{3}\left(n^{3}-3n^{2}+2n\right),\mathrm{and}\\ E\left(X^{k+1}\right) & =pq\cdot \frac{d\left(E\left(X^{k}\right)\right)}{dp}+npE\left(X^{k}\right)\mathrm{for}\;k=3,4,\ldots . \end{array}$$

Given that $N_{1}$ and $N_{2}$ are binomially distributed, we obtain, using the first two terms in the expansion of the log function,

$$\begin{array}{cc} E\left(\hat{ent_{N_{2}}}\right)= & E\left(-\frac{X}{N}\log_{2}\frac{X}{N}\right)\\ = & E\left[-\frac{X}{N}\left(\frac{X-N}{N}-\frac{1}{2}{\left(\frac{X-N}{N}\right)}^{2}\right)\right]\\ = & E\left[-\frac{2X^{2}}{N^{2}}+\frac{3X}{2N}+\frac{X^{3}}{2N^{3}}\right]. \end{array}$$

Now, using the formulae for $E(X^{k})$, it is easy to get

$$E\left(\hat{ent_{N_{2}}}\right)=\left(\frac{1}{2N^{2}}-\frac{2}{N}+\frac{3}{2}\right)p+\left(-\frac{3}{2N^{2}}+\frac{7}{2N}-2\right)p^{2}+\left(\frac{1}{N^{2}}-\frac{3}{2N}+\frac{1}{2}\right)p^{3}.$$

Then, the bias of entropy for $N_{2}$ can be calculated as

$$\begin{array}{cc} bias_{N_{2}}= & E\left(\hat{ent_{N_{2}}}\right)-E\left(ent_{N_{2}}\right)\\ = & E\left(\hat{ent_{N_{2}}}\right)-\left(-2p^{2}+\frac{3}{2}p+\frac{1}{2}p^{3}\right)\\ = & \left(\frac{1}{2N^{2}}-\frac{2}{N}\right)p+\left(-\frac{3}{2N^{2}}+\frac{7}{2N}\right)p^{2}+\left(\frac{1}{N^{2}}-\frac{3}{2N}\right)p^{3}. \end{array}$$

Similarly, the bias for $N_{1}$ is

$$bias_{N_{1}}=\left(\frac{1}{2N^{2}}-\frac{2}{N}\right)\left(1-p\right)+\left(-\frac{3}{2N^{2}}+\frac{7}{2N}\right){\left(1-p\right)}^{2}+\left(\frac{1}{N^{2}}-\frac{3}{2N}\right)p^{3},$$

so the bias for the root node is

(2) $$\begin{array}{cc} bias_{N}= & bias_{N_{1}}+bias_{N_{2}}\\ = & \left(\frac{1}{2N^{2}}-\frac{2}{N}\right)+\left(-\frac{3}{2N^{2}}+\frac{7}{2N}\right)\left(1-2p+2p^{2}\right)+\left(\frac{2}{N^{2}}-\frac{3}{N}\right)p^{3}. \end{array}$$

For the root node, the expected entropy information is $E(\hat{ent})$ for *N* observations. After splitting the root node, it is easy to get the left child node and the right child node with $N_{L}$ observations and $N_{R}$ observations, respectively. Two cases where *X* and *Y* are independent and when they are associated are considered as the following.

**Case 1: Explanatory Variable *X* is Independent of Response Variable *Y*.**

In this case,

$$\begin{array}{cc} E\left(\hat{▵ent}\right)= & E\left(\hat{ent}\right)-\frac{N_{R}}{N}E\left(\hat{ent_{R}}\right)-\frac{N_{L}}{N}E\left(\hat{ent_{L}}\right)\\ = & bias_{N}+E\left(ent\right)\\ \; & -\frac{N_{R}}{N}\left(bias_{R}+E\left(ent_{R}\right)\right)-\frac{N_{L}}{N}\left(bias_{L}+E\left(ent_{L}\right)\right). \end{array}$$

Since *X* is independent of *Y*, so $E\left(ent\right)=E\left(ent_{R}\right)=E\left(ent_{L}\right)$, and

$$\begin{array}{cc} E\left(\hat{▵ent}\right)= & bias_{N}-\frac{N_{R}}{N}bias_{N_{R}}-\frac{N_{L}}{N}bias_{N_{L}}\\ = & \left(\frac{2}{N}+\frac{1}{2N^{2}}-\frac{1}{2N_{L}N_{R}}\right)p+\left(-\frac{3}{2N^{2}}+\frac{3}{2N_{L}N_{R}}-\frac{7}{2N}\right)p^{2}\\ \; & +\left(\frac{3}{2N}+\frac{1}{N^{2}}-\frac{1}{N_{L}N_{R}}\right)p^{3}+\left(\frac{2}{N}+\frac{1}{2N^{2}}-\frac{1}{2N_{L}N_{R}}\right)\left(1-p\right)\\ \; & +\left(-\frac{3}{2N^{2}}+\frac{3}{2N_{L}N_{R}}-\frac{7}{2N}\right){\left(1-p\right)}^{2}\\ \; & +\left(\frac{3}{2N}+\frac{1}{N^{2}}-\frac{1}{N_{L}N_{R}}\right){\left(1-p\right)}^{3}. \end{array}$$

As *X*, *Y* are independent, the split in *X* can be anywhere. It is assumed to be in the middle of *X*, so $N_{L}=N_{R}=\frac{N}{2}$. The other circumstances can be explored in future work. Then we have

$$\begin{array}{cc} E\left(\hat{▵ent}\right)= & \left(\frac{2}{N}-\frac{3}{2N^{2}}\right)p+\left(\frac{9}{2N^{2}}-\frac{7}{2N}\right)p^{2}+\left(\frac{3}{2N}-\frac{3}{N^{2}}\right)p^{3}+\\ \; & \left(\frac{2}{N}-\frac{3}{2N^{2}}\right)\left(1-p\right)+\left(\frac{9}{2N^{2}}-\frac{7}{2N}\right){\left(1-p\right)}^{2}+\\ \; & \left(\frac{3}{2N}-\frac{3}{N^{2}}\right){\left(1-p\right)}^{3}. \end{array}$$

Since $E\left(▵ent\right)=0$, then

$$bias=E\left(\hat{▵ent}\right).$$

If p=0.5, then $bias=\frac{5}{8N}$. This shows that, when *X* and *Y* are independent, as sample size *N* decreases, entropy gain increases. Noise (redundant) variables can be seen as *X* here as they are independent with *Y*. Suppose the number of missing values is $n_{X}$, then the sample size of *X* with missing values becomes $N-n_{X}$. A higher $n_{X}$ means a lower $N-n_{X}$ for fixed *N*. For $bias=\frac{5}{8(N-n_{X}}$, more missing values means a bigger bias for this noise variable *X*, thus with a bigger entropy real gain. In that case noise (redundant) variables with more missing values have a better chance to be chosen.

**Case 2: Explanatory Variable *X* is Associated with Response Variable *Y*.**

In practice, if *X* is not a noise variable, then *X* and *Y* are associated. For example, *X* and *Y* are related as Y=a+bX, where *a* and *b* are constants. Since *X* is dependent on *Y*, the split should be at the same place as that in *Y*. In that case, the sample will become pure after splitting, which means $E\left(\hat{ent_{R}}\right)=E\left(\hat{ent_{L}}\right)=0$. Then, the expectation of entropy gain is:

$$\begin{array}{cc} E\left(\hat{▵ent}\right)= & E\left(\hat{ent}\right)-\frac{N_{R}}{N}E\left(\hat{ent_{R}}\right)-\frac{N_{L}}{N}E\left(\hat{ent_{L}}\right)\\ = & bias_{N}+E\left(ent\right). \end{array}$$

Then, the bias of the entropy gain is

$$\begin{array}{cc} bias= & bias_{N}+E\left(ent\right)-E\left(ent\right)\\ = & bias_{N}. \end{array}$$

Similarly, when p=0.5, from Equation (2), $bias_{N}=-5/\left(8N\right)<0$. So, there are circumstances, when *X* is not a noise variable, and *X*, *Y* are dependent, that we have a negative bias. It is opposite to the situation for independent variables. For $bias=-\frac{5}{8(N-n_{X}}$, more missing values means a smaller bias for this informative variable *X*, thus with a smaller entropy real gain. In that case, informative variable *X* with more missing values has less chance to be chosen.

The approximation is verified by simulation, choosing p=0.5,0.6,...,0.9 as *p* and 1−p are symmetric. For a specific *N* (the total number of observations), $N_{2}∼B\left(N,p\right)$ and $N_{1}=N-N_{2}$ are chosen. Then, the entropy bias in the simulation can be calculated using *N*, $N_{1}$, $N_{2}$ and assumptions from the above two situations.

**Figure 1:**
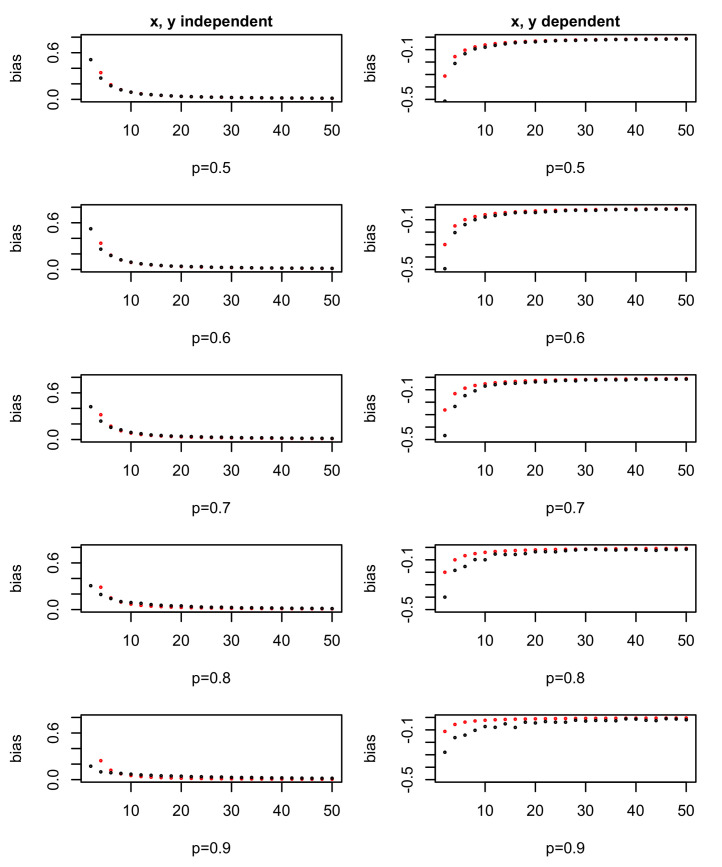
Entropy gain bias in theory and practice. Red dots are theoretical values and black dots are based on simulation. The x axis shows the total number of observations, *N*.

The results in Figure 1 show that the theoretical values are roughly the same as the simulated ones, which confirm our approximation. When *N* gets bigger, the practice entropy gain is almost the same as the theoretical entropy gain, thus resulting with an almost 0 bias value. One difference is that when *N* is small and *p* or 1−p is small, the log approximation used in Equation (1) is not so suitable, so there is gap between the simulated and theoretical results.

For noise variables, the more missing values there are, the bigger the chance they have of being chosen as a splitting variable. For informative variables, the more missing values there are, the smaller chance they have to be chosen. Both situations will lead to bad results. That is why we deal with missing values and other outliers in the data cleaning process in real application.

### 2.2. Bias Related to More Values or Categories

In this section, how the entropy and Gini criteria have bias related to the number of categories or number of possible values in *X* is explored. A $\chi^{2}$ statistic is also involved as a criterion for comparison, which does not have this bias due to more values or categories as its degree of freedom changes accordingly.

The ground truth is assumed as that *X* and *Y* are independent. When the ground truth is unknown, for any split in *X*, the event that *X* is dependent on *Y* in each child node is accepted with probability *p*. The hypotheses are
$H_{0}$: *X* is independent of *Y*;    $H_{1}$: *X* is dependent on *Y*
When $H_{0}$ is true, then *X* is independent of *Y* for any possible split in *X*. The corresponding probability to accept $H_{0}$ is

$${\left(1-p\right)}^{r}$$

where

$$r=\left\{\begin{array}{cc} m-1, & \mathrm{ordered}\;\mathrm{variable}\;X\\ 2^{m-1}-1, & \mathrm{categorical}\;\mathrm{variable}\;X, \end{array}\right.$$

and *m* is the number of unique values for an ordered variable or categories for a categorical variable. When $H_{1}$ is accepted, we have

$$P\left(H_{1}\;\mathrm{is}\;\mathrm{accepted}|H_{0}\;\mathrm{is}\;\mathrm{true}\right)=1-{\left(1-p\right)}^{r},$$

which means that there is at least one split in *X* that makes *X* depend on *Y*. It is easy to see that explanatory variables with more values or categories have a better chance to be chosen even though *X* is independent of *Y*. For the Gini index or entropy gain, they have not eliminated this multiple comparison effect, so they still have that kind of bias. However, for a Chi-squared test [21], it uses the corresponding *p* value instead, and it has different distribution for different degrees of freedom calculated from the possible values or categories in *X*, so it eliminates this effect.

**Figure 2:**
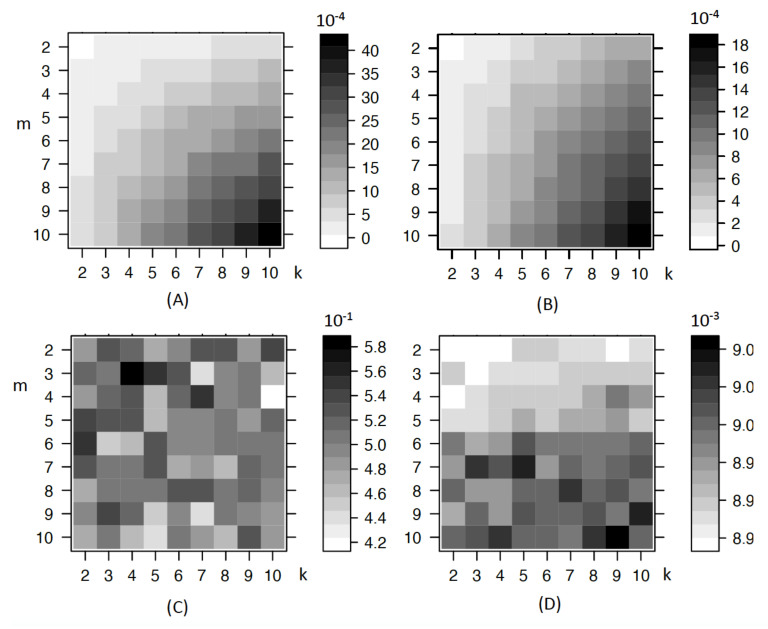
Bias when number of categories or values in *X* and *Y* changes. The four subfigures are the bias of $entropy\;\;gain\left(A\right),\;\;entropy\;\;gain\;\;rate\left(B\right),\;\;Gini\;\;gain\left(C\right),\;\;p{\left(\chi^{2}\right)}_{m,k}\left(D\right)$, respectively, at top left, top right, bottom left and bottom right. Here, the x axis label *k* is the number of values or categories in response variable *Y* and the y axis label *m* is the number of values or categories in explanatory variable *X*. The darker the shade, the higher the bias.

A simulation is conducted to explore the bias effect for the Gini gain and entropy gain while compared with $p\left(\chi^{2}\right)$. The corresponding results are shown in Figure 2. It is obvious, for entropy gain and entropy gain rate, that the bias increases when *k* or *m* increases. For the Gini index, it also increases, but the bias value changes little, being around 0.42 to 0.58. For $\chi^{2}$, as expected, there is no sign of bias due to more values or categories in *X* and *Y*.

In Figure 2, there is some kind of bias trend for Gini index and $p{\left(\chi^{2}\right)}_{m,k}$, but it is clear that they do not show such obvious trend as that of entropy gain and gain rate. For Gini gain, there are also small bias when *k* is 7 and *m* is 3. So, the trend is also not stable when *k* and *m* changes. The range of their bias values are shown at the right side of each sub figure. The important point is how the intensity changes across *k* and *m* in each sub figure. The comparative intensity of the same *k* and *m* among different figures is also important but it is not included in our analysis context. So the values are not scaled. Both entropy gain and entropy gain rate have an obvious trend when *k* and *m* increases, as the color gets darker. However, Gini gain and $p{\left(\chi^{2}\right)}_{m,k}$ do not show such obvious trend. That explains why Gini gain is better than entropy. For classification purposes, the Gini index is chosen as the splitting criterion as its bias due to more values or categories is not that large compared to entropy. The rpart package [23] in R includes the choice of Gini index as the default splitting criterion. For $\chi^{2}$, although it is good, the CHAID package in R can only be applied to categorical variables while our later analysis includes continuous response variables. There are many algorithms to build classification trees, including ID3 [24], C4.5 [25] and CART [14], etc. ID3 is one of the original algorithms, which uses the entropy information criterion, but it does not apply any pruning nor does it deal with numeric attributes or missing values. As an evolution of ID3, C4.5 uses the entropy information gain ratio as the splitting criterion. The splitting ceases when the number of instances to be split is below a certain threshold, and error-based pruning is performed after the growing phase. Further, C4.5 can handle numeric attributes. In terms of CART, such binary trees are constructed based on the Gini index or twoing criterion and the tree is pruned by complexity criterion. It can also involve misclassification costs and prior probability distributions in the tree building process [26]. As software R is used for coding, and the decision tree package rpart is generally based on CART, so CART is chosen as the classification tree using Gini index as the splitting criterion.

## 3. Influence of Noise Variables on CART Computational Complexity

The contribution in this section is to explore how the number of noise variables influences the computational time under simplified conditions using the existing Bonferroni multiplier [27].

This section explores how the number of noise variables influences the computational complexity compared to merely using informative variables. The term computational complexity here refers to the time complexity of an algorithm. In computer science, the time complexity of an algorithm quantifies the amount of time taken by an algorithm to run as a function of the length of the string representing the input. Time complexity is commonly estimated by counting the number of elementary operations (such as addition, subtraction, multiplication, division, comparison operations) performed by the algorithm, where an elementary operation takes a fixed amount of time to perform. Thus, the amount of time taken and the number of elementary operations performed by the algorithm differ by at most a constant factor. In that way, the number elementary operation is counted to represent the computational complexity.

For CART, the following ideal conditions are assumed:

1. All the independent variables can be divided into effective variables and noise variables. The criterion is whether they are used in the tree growing process or not. As the most effective variables will be chosen for splitting firstly. Those variables not chosen have less effect than those chosen. A tree building process includes both a growing process and pruning process (or stopping criteria). This time, the tree is assumed to choose the stopping criteria, so that we only need to concentrate on the growing process. Noise variables refer to variables that are not used in the tree growing process.
2. All variables are categorial variables for convenience of calculation.
3. For every split, no matter how many categories the independent variable has, there are always two child nodes after the parent node since CART is a binary tree. All nodes are assumed to stop splitting at the same time which means the depth is the same for every branch on the same level.
4. When one independent variable is chosen as a split, it will not be chosen again.

Such simplifying assumptions are made for easy of calculation. In reality, the process is more complex than that. Define *N* as the number of explanatory variables including both effective variables and noise variables, *M* as the number of effective variables, and $c_{j}$ as the number of categories in the *j*th independent variable. In the splitting process, the explanatory variable will be split into two intervals (numerical) or groups (categorical). The number of all possible ways of separating the $c_{j}$ categories into two groups is the Bonferroni multiplier [27]. Here since all categories are split into two groups, it is

$$S\left(c_{j},2\right)=\sum_{r=1}^{2}{\left(-1\right)}^{2-r}\frac{r^{c_{j}}}{r!\left(2-r\right)!}.$$

### 3.1. Computational Complexity without Noise Variables

For the initial split, assume variable $a_{1}$ is chosen, and the computational complexity is

$$2^{0}\sum_{j=1}^{M}S\left(c_{j},2\right)b+m,$$

where *b* is the computational complexity involved in calculating the entropy information for one possible split in one variable and *m* is the computational complexity for calculating the entropy information in *y*.

After that, variable $a_{1}$ will not be used again because of Assumption 4. Assume variable $a_{2}$ is chosen as the split for both child nodes after $a_{1}$, and the computational complexity for both child nodes are similar, so the total computational complexity at step 2 is

$$2^{1}\sum_{j=2}^{M}S\left(c_{j},2\right)b.$$

Even though it is essential to calculate the entropy gain from the parent node to child nodes, just calculating the entropy information in child nodes is sufficient since the parent node entropy information has already been calculated from the previous step. So here we just count the computational complexity for the child nodes.

Under Assumption 3, the number of terminal nodes increases in a power of 2. After summing all the computational complexity for all the nodes, the computational complexity for the whole tree is:

$$CC_{effect}=\sum_{s=0}^{M-1}2^{s}\sum_{j=s+1}^{M}S\left(c_{j},2\right)b+m.$$

### 3.2. Computational Complexity with Noise Variables

It is easy to calculate the computational complexity with noise variables in a similar way to the case without noise variables. The difference is the total number of explanatory variables in use is not *M* but *N*, which includes the noise variables. The difference comparison will be shown in Section 3.3. For the initial split, assume variable $a_{1}$ is chosen, so the computational complexity is

$$2^{0}\sum_{j=1}^{N}S\left(c_{j},2\right)b+m.$$

For the second split, it is

$$2^{1}\sum_{j=2}^{N}S\left(c_{j},2\right)b.$$

There are many reasons for the tree to stop growing, such as the node becomes pure or all the variables have the same proportion in all the *y* categories. At level M+1, all the *M* effective variables are used, so the tree will test whether the first noise variable is effective or not. Since noise variables are assumed to be those not selected by the tree. So after the testing, the tree will stop growing. The computational complexity for the testing is

$$2^{M}\sum_{j=M+1}^{N}S\left(c_{j},2\right)b.$$

For the whole tree, the computational complexity is

$$CC_{effect+noise}=\sum_{s=0}^{M}2^{s}\sum_{j=s+1}^{N}S\left(c_{j},2\right)b+m.$$

### 3.3. Computational Complexity Increase

The increase in computational complexity due to the presence of noise variables is

$$\begin{array}{cc} CC_{inc}= & CC_{effect+noise}-CC_{effect}\\ = & \sum_{s=0}^{M}2^{s}\sum_{j=s+1}^{N}S\left(c_{j},2\right)b+m-\sum_{s=0}^{M-1}2^{s}\sum_{j=s+1}^{M}S\left(c_{j},2\right)b-m\\ = & \sum_{s=0}^{M}2^{s}\sum_{j=M+1}^{N}S\left(c_{j},2\right)b. \end{array}$$

Assuming that the $c_{j}$ has the same value across different *j*, then we can rewrite $S\left(c_{j},2\right)b$ as one value *u*. Then $CC_{inc}$ becomes

$$CC_{inc}=\left(2^{M+1}-1\right)\cdot \left(N-M\right)u.$$

which is a linear function of the number of noise variables, N−M. So, even when methods which increase the dimension of explanatory variables are used before the application of decision trees, the computational complexity will not increase dramatically.

## 4. Conclusions

For trees, there are many splitting criteria to choose. We explored their splitting bias due to missing values, variables with more values or categories. Results show that noise variables with more missing values have a better chance to be chosen, but informative variables with more missing values have a less chance to be chosen. Between entropy information and the Gini index, we choose the latter as the splitting criterion as its bias due to more values or categories is not that obvious compared to the former as shown in Figure 2. Under some assumptions, we studied the influence of noise variables on CART computational complexity. That increase will generally only result in a linear increase in the computational complexity.

The limitations of the research are that the analysis is conducted under simple assumptions, more complex assumptions are suggested in the future research. For example, the theoretical result under totally independent or totally dependent are conducted but those between them are analyzed by simulation instead of by theory. In the computational complexity section, future research can be done under less condition limitations.

## Data Availability

Not applicable.
